# Identification of Retinoic Acid in a High Content Screen for Agents that Overcome the Anti-Myogenic Effect of TGF-Beta-1

**DOI:** 10.1371/journal.pone.0015511

**Published:** 2010-11-30

**Authors:** Chateen Krueger, F. Michael Hoffmann

**Affiliations:** McArdle Laboratory for Cancer Research, Departments of Oncology and Medical Genetics, School of Medicine and Public Health, University of Wisconsin-Madison, Madison, Wisconsin, United States of America; Fundação Oswaldo Cruz, Brazil

## Abstract

**Background:**

Transforming growth factor beta 1 (TGF-β1) is an inhibitor of muscle cell differentiation that is associated with fibrosis, poor regeneration and poor function in some diseases of muscle. When neutralizing antibodies to TGF-β1 or the angiotensin II inhibitor losartan were used to reduce TGF-β1 signaling, muscle morphology and function were restored in mouse models of Marfan Syndrome and muscular dystrophy. The goal of our studies was to identify additional agents that overcome the anti-myogenic effect of TGF-β1.

**Methodology/Principal Findings:**

A high-content cell-based assay was developed in a 96-well plate format that detects the expression of myosin heavy chain (MHC) in C2C12 cells. The assay was used to quantify the dose-dependent responses of C2C12 cell differentiation to TGF-β1 and to the TGF-β1 Type 1 receptor kinase inhibitor, SB431542. Thirteen agents previously described as promoting C2C12 differentiation in the absence of TGF-β1 were screened in the presence of TGF-β1. Only all-trans retinoic acid and 9-cis retinoic acid allowed a maximal level of C2C12 cell differentiation in the presence of TGF-β1; the angiotensin-converting enzyme inhibitor captopril and 10 nM estrogen provided partial rescue. Vitamin D was a potent inhibitor of retinoic acid-induced myogenesis in the presence of TGF-β1. TGF-β1 inhibits myoblast differentiation through activation of Smad3; however, retinoic acid did not inhibit TGF-β1-induced activation of a Smad3-dependent reporter gene in C2C12 cells.

**Conclusions/Significance:**

Retinoic acid alleviated the anti-myogenic effect of TGF-β1 by a Smad3-independent mechanism. With regard to the goal of improving muscle regeneration and function in individuals with muscle disease, the identification of retinoic acid is intriguing in that some retinoids are already approved for human therapy. However, retinoids also have well-described adverse effects. The quantitative, high-content assay will be useful to screen for less-toxic retinoids or combinations of agents that promote myoblast differentiation in the presence of TGF-β1.

## Introduction

Transforming growth factor beta 1 (TGF-β1) plays a prominent role in regulating a variety of cellular functions including cell migration, cell proliferation, apoptosis, differentiation, immunosuppression, inflammation, tumor-suppression, and angiogenesis [1], [2]. It has long been recognized that the specific cellular response to TGF-β1 is context dependent and varies according to the cell type, the cellular environment and the activity of other signaling pathways [3]. Elevated TGF-β1 has been associated with several disease states including metastasis and immune evasion by cancer cells, and fibrosis in many tissues including skin, lung and kidney [4], [5].

One of the earliest cellular responses reported for TGF-β1 was inhibition of myoblast differentiation in culture [6], [7]. TGF-β1 inhibits expression of two key transcriptional mediators of muscle cell differentiation, MyoD and myogenin [8]. The TGF-β1 activated protein Smad3 binds directly to the MyoD bHLH domain to block MyoD/E protein dimerization and DNA binding [9]. Smad3 also binds to and interferes with the myogenic transcription factor MEF2 to prevent muscle-specific gene expression [10]. In contrast, increased expression of the inhibitory Smad, Smad7, promotes myogenesis [11]. Another TGF-β family member, myostatin, is also a potent inhibitor of muscle differentation and growth [12], [13].

The anti-myogenic role of TGF-β1 has been associated with muscle disease. For example, TGF-β1 levels are elevated in dystrophic and injured muscle [14], [15]. In injured muscle, TGF-β1-induced myofibroblasts cause excessive fibrosis [16], [17], [18], [19]. Recently, Cohn, Dietz, and colleagues reported that the elevated TGF-β1 signaling in the muscles of mouse models of Marfan syndrome (MFS) and muscular dystrophy contributed to the failure of muscle regeneration [20]. MFS is an autosomal dominant disorder caused by mutations in the gene encoding fibrillin-1. Fibrillin-1 negatively regulates TGF-β1 activation and signaling. Fibrillin-1 mutant mice have decreased muscle fiber size and number associated with increased levels of the active signaling intermediates of TGF-β1 signaling, phospho-Smad2 and phospho-Smad3 [20]. Elevated levels of nuclear-localized activated Smads were also detected in skeletal muscle from X-linked muscular dystrophic (mdx) mutant mice, even in the absence of myostatin [20]. Fewer proliferating satellite cells, the cells responsible for muscle regeneration [21], [22], were detected in the muscle of fibrillin-1 mutant mice, suggesting that TGF-β1 might exert its effect by inhibiting satellite cell proliferation and differentiation. Reduced satellite cell function is also associated with poor muscle regeneration in muscular dystrophy [23]. Interestingly, spikes of elevated TGF-β1 expression and phospho-Smads occur in wildtype muscle after damage by injection of a snake venom cardiotoxin, but these increases were not detected 18 days after injury in wildtype mice. In contrast, the increases were maintained in the skeletal muscle of the fibrillin-1 mutant mice, suggesting that the prolonged increase in TGF-β1 expression impedes regeneration.

Treatment of the fibrillin-1 mutant mice or the mdx mutant mice with TGF-β1-neutralizing antibody or the angiotensin II type 1 receptor inhibitor losartan improved muscle architecture, repair and function [20]. Both TGF-β1-neutralizing antibody and losartan treatment of 9-month-old mdx mice improved muscle regeneration assayed four and eighteen days after snake toxin-induced injury of muscle from mdx mice, e.g., increased neonatal myosin-positive fibers were detected. Importantly, long term (6–9 months) administration of losartan to mdx mice beginning at 6 weeks, provided detectable improvement in the diaphragm and gastrocnemius muscles with decreased evidence of fibrosis. In behavioral tests, treated mdx mice had increased grip strength and reduced muscle fatigue compared to the placebo-treated mdx mice. Losartan treatment led to increased muscle mass and force generation in the extensor digitorum longus muscles correlating with an increase in the number of muscle fibers [20]. ln addition to MFS and muscular dystrophy, losartan is also known to inhibit TGF-β1 signaling in other disease states such as renal disease and cardiomyopathy [24], [25]. Studies in animal models of kidney fibrosis have reported enhanced efficacy by combining losartan with TGF-β inhibitors [26], [27], [28].

Inhibition of TGF-β signaling has focused on disruption of the ligand-receptor interaction with neutralizing antibodies or by inhibition of the receptors using small molecule inhibitors of the receptor kinase activity [29]. TGF-β1 signaling is effectively inhibited by small molecule compounds targeted to the TGF-β Type 1 receptor kinase, Alk5 [30]. One such compound, SB431542, blocks the increased phosphorylation of Smad2 caused by myostatin activation of the TGF-β Type 1 receptor kinases in C2C12 myoblasts [31]. Treatment with 10 µM SB431542 led to the formation of myotubes with an enlarged diameter upon differentiation of C2C12 cells *in vitro*. However, prolonged treatment (96 hours) with 10 µM SB431542 led to decreased expression of myosin heavy chain in the C2C12-derived myotubes; the inhibitor also caused an increase in the cross-sectional area of isolated iliofibularis skeletal muscle fibers from *Xenopus laevis*, but without a concomitant increase in tetanic force, leading to the conclusion that SB431542 causes a nonfunctional hypertrophy in muscle fibers that may not be of therapeutic benefit [31]. Targeting the TGF-β receptor may be undesirable because TGF-β plays important roles in normal physiology including tumor suppression, wound healing, or inflammation. Consistent with this concern, recent clinical trial results indicate that agents directly targeting the TGF-β receptor may have adverse effects [32], [33].

Given the clear evidence for elevated TGF-β1 signaling in myopathy and the reported concerns associated with blocking TGF-β1 signaling at the level of the receptor kinase, we sought to develop a quantitative cell-based screen for agents that overcome the anti-myogenic effect of TGF-β1 on the muscle satellite cell line C2C12. We evaluated several agents in the assay for their ability to overcome the TGF-β1 inhibition of muscle differentiation. Inhibition of the TGF-β1 Type 1 receptor kinase with SB431542 allowed C2C12 differentiation in the presence of TGF-β1 as expected. Of the other 13 agents tested, only all-trans retinoic acid and 9-cis retinoic acid overcame the TGF-β1 effect completely. Neither of the retinoic acid compounds prevented TGF-β1 activation of the Smad3-dependent reporter gene SBE12-lux in C2C12 cells, indicating that their ability to overcome the anti-myogenic effect of TGF-β1 is likely through a Smad3-independent mechanism.

## Materials and Methods

### Cell culture

Mouse myoblast C2C12 cells were obtained from the American Type Culture Collection (CRL-1772) and cultured in growth media consisting of 10% fetal bovine serum in Dulbecco's Modified Eagle's Medium (DMEM). To induce differentiation, cells were washed one time in 2% horse serum (HS)/DMEM (Invitrogen, Carlsbad, CA) in DMEM and cultured in 2% HS/DMEM for 96 hours [34].

### Differentiation Assay

Cells were plated at 7,500 cells per well in black 96-well optical plates (BD Biosciences, San Jose, CA) and allowed to adhere for one day. The next day, cells were washed one time in 2% horse serum/DMEM, and cultured +/− TGF-β1 (R & D Systems, Minneapolis, MN) and +/− candidate differentiation agent. After 4 more days in culture, cells were fixed in 4% paraformaldehyde in phosphate-buffered saline (PBS) for 15 minutes, permeabilized in 0.5% Triton X-100 in PBS for 5 minutes, and blocked in 3% bovine serum albumin (BSA) in PBS for 30 minutes. The antibody MF-20 (Developmental Studies Hybridoma Bank, Iowa City, IA), diluted to 5 µg/mL in 3% BSA/PBS, was used to detect myosin heavy chain (MHC). To remove unbound primary or secondary antibody, 3 five-minute washes were performed in 0.05% Triton X-100/PBS with a final wash in PBS. Incubation with the secondary antibody, Alexa Fluor 594 goat anti-mouse IgG (Cat. No. A-11032, Invitrogen) diluted to 7 µg/mL in 3% BSA/PBS, was used to detect MF-20. Co-incubation of Hoechst 33342 (Invitrogen) (diluted to 7 µg/mL in 3% BSA/PBS) with the secondary antibody was used to stain the nuclei. All antibody and nuclear stain incubations were performed at room temperature for 1 hour.

### Image acquisition and analysis

Three non-contiguous images from each well were obtained using a high-content imager, the BD Pathway 855 (BD Biosciences, San Jose, CA). Images were obtained of both nuclei and cytoplasmic regions using a 10× objective. Image analysis was performed using BD AttoVision software v.1.6. A segmentation strategy, Ring 2 Outputs Band, was used to identify and count each nucleus in the Hoechst channel and the respective Alexa 594-stained cytoplasmic band was quantified by dialing out from each nucleus by a fixed distance. Image Data Explorer was used to determine the total number of nuclei, the average pixel intensity of each cytoplasmic band, as well as a percent muscle differentiation value. To determine how many cells were positive for myosin heavy chain, an intensity threshold of 350–450 pixels was set to count the nuclei whose cytoplasmic bands were positive for Alexa Fluor 594 over background. The data were transformed into the percent of nuclei surrounded by MHC expression. Percent muscle differentiation was obtained by dividing the number of nuclei positive for MHC by the total number of nuclei and multiplying by 100. This approach avoided artifacts due to well-to-well variation in overall staining intensity. Data were plotted using XLfit 5.2 (IDBS, Alameda, CA) or Microsoft Excel. The effective dose producing a 50% response (ED50) or the half maximal inhibitory concentration (IC50) was calculated using XLfit 5.2. Agents screened: All-trans retinoic acid (atRA), 9-cis retinoic acid (9-cisRA), epigallocatechin-3-gallate (EGCG), ascorbic acid, tranilast, losartan potassium, captopril, resveratrol, 1α,25-dihydroxyvitamin D_3_ (vitamin D) and PD123,319 were purchased from Sigma-Aldrich (St. Louis, MO). TGF-β1 and Relaxin-2 were obtained from R & D Systems. SB431542 was obtained from Tocris Bioscience (Ellisville, MO). Estrogen was a gift from Dr. Wei Xu (UW-Madison). R1881 was a gift from Dr. George Wilding (UW-Madison). 17-Dimethylamino-ethylamino-17-demethoxygeldanamycin (17-DMAG) was a gift from Dr. Shannon Kenney (UW-Madison). Specific inhibitor of Smad3 (SIS3) was purchased from EMD Chemicals (Gibbstown, NJ). All experiments were conducted with 0.1% or less of DMSO (Sigma-Aldrich). L-ascorbic acid (Sigma-Aldrich) at a concentration 1 µM was added to experimental samples containing EGCG in order to increase its stability and minimize oxidation.

### Reporter Gene Assays

C2C12 cells were plated at a density of 20,000 cells per well in 24-well plates. 24 hours later, cells were transfected with a Smad binding element 12 (SBE 12)-luciferase reporter gene and a β-galactosidase construct with transfection reagent TransIT-LT1 (Mirus Bio, Madison, WI). After 24 hours, cells were treated with a DMSO control, 1 µM SB431542, and 50 pM TGF-β1 with or without different concentrations of 9-cis retinoic acid and all-trans retinoic acid, in triplicate. After 24 hours, cells were lysed with the Galacto-Star β-Galactosidase Reporter Gene Assay System for Mammalian Cells (Applied Biosystems, Calsbad, CA). Luciferase levels were determined using Bright-Glo luciferase reagent (Promega, Madison, WI) and β-galactosidase levels were measured with the Galacto-Star Kit. Luminescence was measured using a Wallac 1420 Victor Plate Reader. Luciferase values were normalized to β-galactosidase levels to account for well-to-well differences in transfection efficiency.

Statistical analysis methods: Data were obtained from a minimum of 3 independent experiments. The average and standard deviations were calculated and are presented on each figure. For Table 1, the statistical significance was assessed using the two-sided Wilcoxon Rank Sum Test provided in the mStat package (http://www.mcardle.wisc.edu/mstat/).

**Table 1:** Compounds tested for their ability to overcome the anti-myogenic effect of TGF-β1 in C2C12 cells.

	Treatment	Dose	% Rescue	p-value
A	TGF-β1, alone	50 pM	0	
B	SB431542	1 µM	100	
C	Losartan	1 µM	−15	
		10 µM	−17	
		25 µM	−1	
		100 µM	−2	
	Captopril	10 µM	1	
		100 µM	8	p<0.01
		500 µM	24	p<0.001
		750 µM	20	p<0.001
		1 mM	14	p<0.001
	PD123,319	10 µM	−4	
		100 µM	−16	p<0.001
D	Tranilast	5 µM	−10	p<0.001
		50 µM	−10	p<0.001
		200 µM	−27	p<0.001
	SIS3	0.3 µM	3	
		1 µM	−4	p<0.05
		3 µM	−6	p<0.001
	17-DMAG	1 nM	−2	
		5 nM	−1	
		10 nM	−7	p<0.05
	Relaxin	1.7 nM	0	
		17 nM	0	
		167 nM	−2	
E	EGCG	50 nM	5	
		500 nM	4	
	Resveratrol	10 nM	3	
		100 nM	5	
		1 uM	−4	
F	R1881	5 nM	−1	
		25 nM	−6	p<0.05
		100 nM	2	
	Estrogen	1 nM	0	
		5 nM	3	
		10 nM	9	p<0.05
G	9-Cis Retinoic Acid	1 nM	10	p<0.001
		25 nM	79	p<0.001
		50 nM	105	p<0.001
		100 nM	129	p<0.001
		250 nM	185	p<0.001
	All-Trans Retinoic Acid	1 nM	10	p<0.01
		25 nM	22	p<0.001
		50 nM	48	p<0.001
		100 nM	71	p<0.001
		250 nM	119	p<0.001

## Results and Discussion

### Detection of C2C12 differentiation by imaging in a 96-well plate format

**Figure 1:**
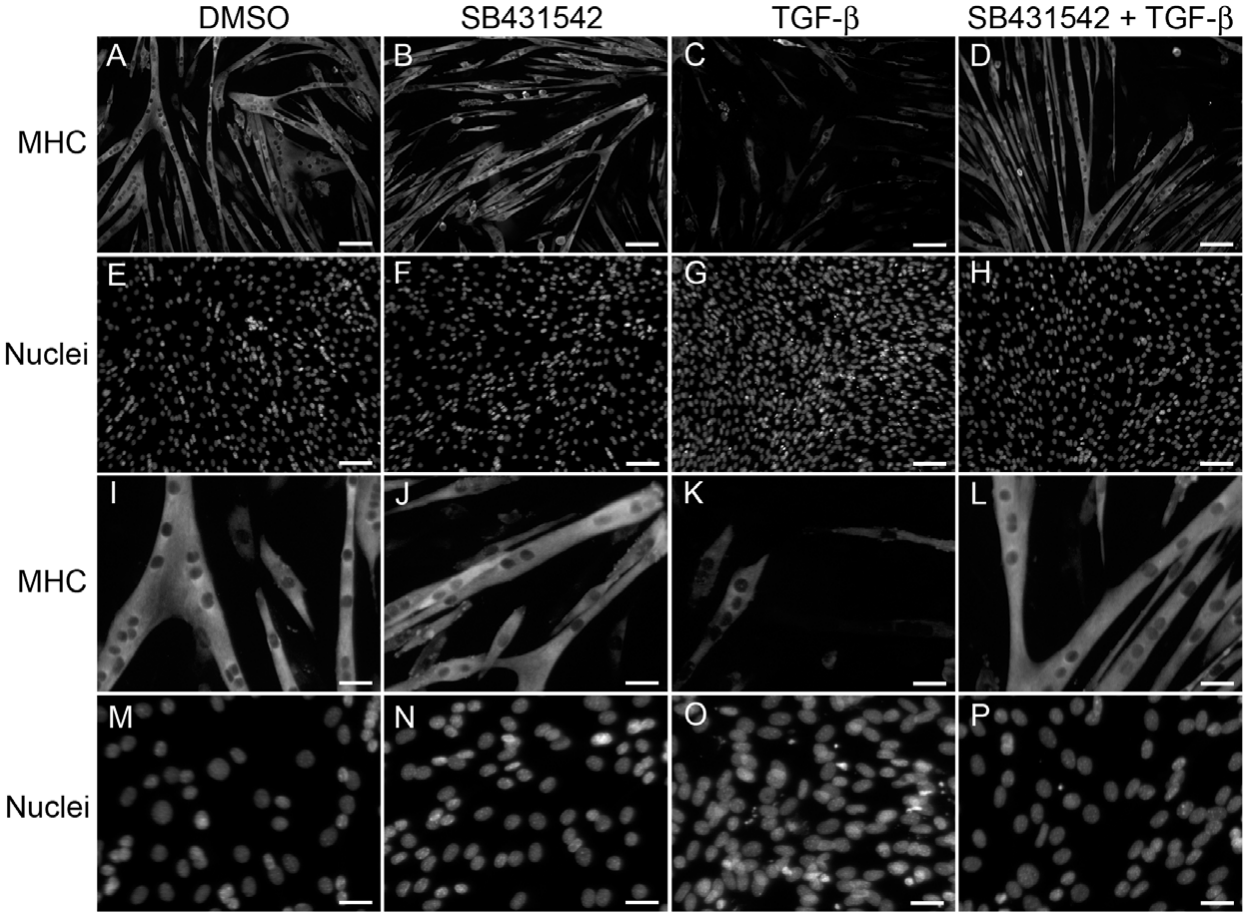
C2C12 muscle differentiation is inhibited by TGF-β1 and restored by SB431542. Cells were treated with vehicle control (DMSO) (A and E), 1 µM SB431542 (B and F), 50 pM TGF-β1 (C and G), or SB431542 and TGF-β1 (D and H). After 96 hours, cells were stained with antibodies against MHC (A–D) or with Hoechst 33342 (E–H). DMSO (0.1%) and SB431542-treated cells were indistinguishable from untreated C2C12 cells (not shown) for differentiation (A, B) or number of nuclei (proliferation) (E, F). TGF-β1 inhibited differentiation as evidenced by reduced staining for MHC (C). Addition of SB431542 with TGF-β1 prevented the TGF-β1-mediated reduction in MHC staining (D). The number of nuclei was increased in TGF-β1-treated cells (G) but was comparable to control levels when SB431542 was also present (H). Images (A–H) show an entire field obtained during BD Pathway image acquisition (scale bar = 100 µm). The bottom 8 panels (I–P) show a 3× enlargement (scale bar = 33 µm) of a region from each of the 8 full field images in A–H.

In order to screen for agents that overcome the anti-myogenic effect of TGF-β1 on C2C12 differentiation, an assay was established that quantified C2C12 cell differentiation as indicated by expression of myosin heavy chain (MHC). To permit the eventual screening of large numbers of compounds, the assay was formatted into 96-well plates and high content imaging technology was used to minimize the number of manipulations and cost of reagents per well and to maximize the efficiency and throughput of screening compounds. Quantification of the percent of nuclei surrounded by MHC-staining was achieved using the high content imaging capabilities and analysis software of the BD Pathway. C2C12 cells were stimulated to differentiate into multi-nucleated, MHC-expressing myotubes in the 96-well plate format by the addition of 2% horse serum in DMEM. After 96 hours, C2C12 cells were stained to detect MHC-expressing myotubes (Figure 1A–1D, 1I–1L) and nuclei (Figure 1E–1H, 1M–1P). Panels 1I–1P show a 3× enlargement of a region from each of the 8 images shown in panels 1A–1H. Vehicle control (DMSO) or SB431542 did not alter differentiation (Figure 1A–1B, 1I–1J) or proliferation (Figure 1E–1F, 1M–1N). TGF-β1 inhibited myoblast differentiation into myotubes (Figure 1C, 1K). Addition of SB431542, a TGF-β Type 1 receptor inhibitor, to the TGF-β1-containing media permitted differentiation (Figure 1D, 1L). The number of nuclei was increased in TGF-β1 treated wells (Figure 1G, 1O) but remained at control levels with TGF-β1 plus SB431542 (Figure 1H, 1P). We noted that differentiation of C2C12 cells in the 96-well plate was sensitive to many variables in the assay including cell passage number, cell density, media conditions and time. For example, at higher cell densities and longer times, MHC expression and myotube formation were readily observed in 10% FBS/DMEM as well as the standard “differentiation media” which contains 2% horse serum in DMEM.

### C2C12 imaging assay allows evaluation of dose-response properties

**Figure 2:**
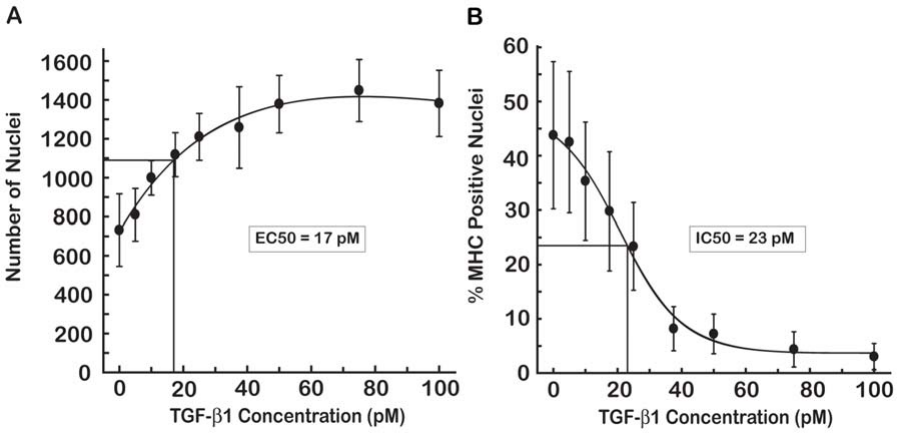
Dose-Response of TGF-β1 on C2C12 Proliferation and Differentiation. C2C12 cells were treated with different concentrations of TGF-β1 for 96 hours and stained for nuclei and MHC. (A) Cell proliferation, as denoted by number of nuclei, increased in a dose-dependent manner with an EC50 for TGF-β1 of 17 pM. (B) Myoblast differentiation, as measured by percentage of nuclei positive for MHC, decreased with increasing concentrations of TGF-β1 with an IC50 of 23 pM.

A dose-response curve of TGF-β1 was performed to determine the potency of TGF-β1 inhibition of C2C12 differentiation (Figure 2). Increasing concentrations of TGF-β1 induced cell proliferation with an ED50 (50% of the maximal increase in the number of nuclei) of 17 pM (Figure 2A). TGF-β1 caused a dose-dependent repression of myoblast differentiation with an IC50 (50% inhibition of MHC positive nuclei) of 23 pM (Figure 2B). For subsequent assays to detect agents that overcome the TGF-β1 effect we used 50 pM TGF-β1; this concentration provided close to maximal repression but might be more sensitive to interference than much higher concentrations of TGF-β1.

**Figure 3:**
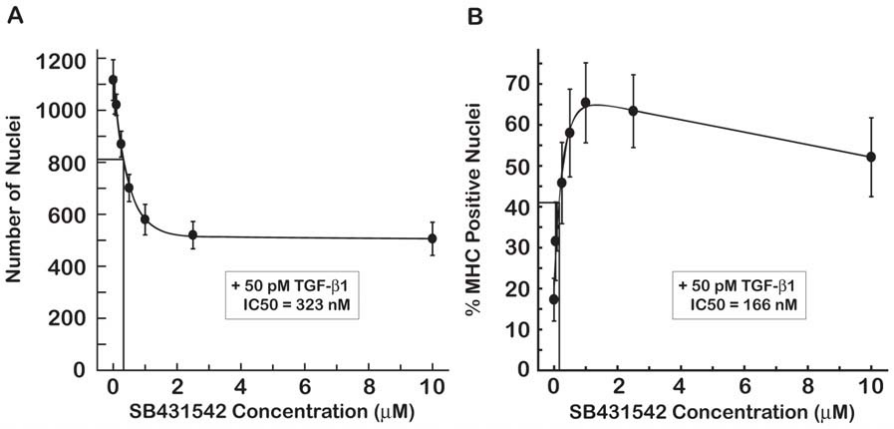
Dose-Response of SB431542 on C2C12 proliferation and differentiation in the presence of TGF-β1. TGF-β1 (50 pM) was used to inhibit C2C12 differentiation. (A) Increasing concentrations of SB431542 caused a decrease in the number of nuclei with an IC50 of 323 nM. (B) SB431542 overcame the TGF-β1 inhibition of C2C12 differentiation with an EC50 of 166 nM. At 10 µM SB431542, we observed a slight but reproducible reduction in the percent of MHC positive nuclei.

The C2C12 cell-based assay also was used to determine the potency of SB431542 on preventing the TGF-β1 anti-myogenic effect. The number of nuclei was reduced approximately two fold by increased concentrations of SB431542 (Figure 3A). The concentration of SB431542 needed to achieve 50% of this two-fold reduction (IC50) was 323 nM. The two-fold reduction in number of nuclei was on the same order as the increase in the number of nuclei observed in the presence of TGF-β1 (Figure 2A), suggesting that SB431542 was preventing the effect of TGF-β1. The percent of MHC positive nuclei was determined in myoblasts treated with 50 pM TGF-β1 and with increasing concentrations of SB431542 (Figure 3B). The concentration of SB431542 needed to restore 50% of the maximal number of MHC positive nuclei (EC50) was 166 nM.

Although several compounds are reported to stimulate C2C12 cell differentiation *in vitro*, there has been no systematic analysis of compounds promoting C2C12 differentiation in the presence of TGF-β1. To identify compounds that prevent the TGF-β1-mediated suppression of myogenesis in the C2C12 imaging assay, compounds were added at several different concentrations to the wells of C2C12 cells at the same time as differentiation media and 50 pM TGF-β1 (Table 1). The number of nuclei and percent MHC positive nuclei were quantified after 96 hours. The percent rescue of differentiation was calculated by subtracting the background, which was assumed to be the percent MHC positive nuclei in the presence of TGF-β1, dividing by the background-subtracted percent MHC positive nuclei in the presence of SB431542+ TGF-β1 and multiplying by 100. This resulted in 0% rescue with TGF-β1 (Table 1A) and 100% rescue for the SB431542+ TGF-β1 (Table 1B). The percent rescue provided by each treatment was evaluated for statistical significance by a two-sided Wilcoxon Rank Sum test. Only significant p-values are shown. Some treatments caused significant but negative rescue percentages, indicating reduced, rather than improved differentiation in the presence of TGF-β1 (Table 1). Our focus was on treatments that significantly improved rescue of differentiation.

### Partial recovery of C2C12 differentiation by the Angiotensin-converting enzyme (ACE) inhibitor captopril

The first compounds to be evaluated in the assay were inhibitors of the angiotensin pathway because the TGF-β1 effect was alleviated in mouse models of MFS and muscular dystrophy by losartan, an angiotensin II type 1 receptor antagonist [20]. We did not observe increased C2C12 differentiation by losartan *in vitro* in the presence of TGF-β1 (Table 1C), which was consistent with a previous report indicating that losartan does not increase MHC expression in C2C12 cells *in vitro* [35]. Two other agents targeted to the angiotensin II pathway, captopril and PD123,319 were also examined. Captopril is an angiotensin converting enzyme (ACE) inhibitor that inhibits the conversion of angiotensin I to angiotensin II. ACE inhibitors are used to treat high blood pressure and help to alleviate the effects of a heart attack and diabetic nephropathy. Captopril has been shown to reduce TGF-β1-induced kidney and colon fibrosis *in vivo* [36], [37]. ACE activity negatively affects C2C12 muscle differentiation *in vitro*, and ACE inhibitors increase MHC expression in differentiating myoblasts and promote myotube formation [35], [38]. We observed a statistically significant (p<0.01) but modest 8% increase in differentiation in the presence of TGF-β1 when 100 µM Captopril was present and an average of 19% rescue with concentrations up to 1 mM (p<0.001) (Table 1C). This is similar to the effective *in vitro* concentration range of Captopril reported previously [35], [38]. PD123,319 is an angiotensin II type 2 receptor antagonist that has been shown to enhance myoblast formation when added at 10 µM and 100 µM to differentiation media [35]. Angiotensin II type 2 receptor antagonists are used to treat diabetes-induced kidney damage, hypertension, and damage from congestive heart failure. However, under the conditions of our assay, PD123,319 was unable to restore C2C12 cell differentiation (Table 1C).

### Four TGF-β signaling inhibitors fail to restore C2C12 differentiation

The TGF-β1 Type 1 receptor kinase inhibitor SB431542 was used as a positive control to establish the assay, so we examined four other reported inhibitors of TGF-β1 signaling: tranilast, specific inhibitor of Smad3 (SIS3), 17-Dimethylamino-ethylamino-17-demethoxygeldanamycin (17-DMAG) and relaxin. Tranilast inhibits TGF-β1-mediated differentiation of bone-derived stromal cells and proliferation of LNCaP and PC-3 prostate cancer cells [39]. It also inhibits proliferation of several mammary carcinoma cell lines as well as TGF-β1-induced phospho-Smad2 [40]. Tranilast was reported to be effective at improving muscle function in BIO14.6 hamsters and mdx mouse models in *vivo* [41]. SIS3, a cell-permeable pyrrolopyridine, inhibits TGF-β1 induced phosphorylation of Smad3, Smad3 binding to Smad4, and Alk-5 induction of p3TP-lux reporter gene activity in scleroderma fibroblasts [42]. SIS3 also has been reported to block Smad3 phosphorylation, myostatin-induced fibrosis and proliferation of muscle fibroblasts [43]. An indirect mechanism for inhibiting TGF-β1 signaling is through targeting Hsp90, a molecular chaperone that plays a role in the proper folding of proteins, the signaling of various protein kinases, and tumorigenesis. The Hsp90 inhibitor geldanamycin is reported to prevent muscle atrophy [44] and recent studies indicate that geldanamycin can inhibit TGF-β1 signaling [45], [46]. The peptide hormone relaxin has been reported to inhibit TGF-β1 signaling in renal fibroblast cell lines in part by preventing Smad2 phosphorylation and translocation to the nucleus [47]. Relaxin restored muscle differentiation in C2C12 cells transfected with a TGF-β1 expression construct [48] and was shown to inhibit TGF-β1 induced collagen synthesis and deposition in myoblasts *in vivo* and *in vitro* [49]. None of these four inhibitors, tranilast, SIS3, 17-DMAG, or relaxin was effective in preventing TGF-β1 inhibition of C2C12 cell muscle differentiation (Table 1D).

### Two inhibitors of oxidative stress fail to restore C2C12 differentiation

Increased oxidative stress exacerbates muscle degeneration in dystrophic muscle [50]. Two natural products that reduce oxidative stress are resveratrol, present in red wine and peanuts [51] and epigallocatechin-3-gallate (EGCG), the primary polyphenol in green tea [52]. In *in vitro* assays, resveratrol reduced skeletal muscle atrophy, enhanced glucose uptake, inhibited NFkB-mediated protein degradation, and improved muscle function [53], [54], [55]. EGCG reduced oxidative stress in C2C12 cells, thereby attenuating the expression of atrogin-1 and MuRF-1, two muscle atrophy-related ubiquitin ligases [52]. EGCG also abrogated the expression of lipofuscin, a marker of oxidative stress, when subcutaneously injected into mdx mice from birth, resulting in improved muscle function, force, and physiology [56]. Neither resveratrol nor EGCG was sufficient to overcome TGF-β1 inhibition of C2C12 cell muscle differentiation (Table 1E).

### Partial recovery of C2C12 differentiation by estrogen

We tested two hormones that are known to augment differentiation of muscle cells and interact with TGF-β1 signaling. Androgens play a pivotal role in muscle function and development and contribute to increased muscle mass and strength [57], [58]. R1881 is a synthetic androgen that has been shown to induce muscle differentiation in androgen receptor-transfected myoblasts [59]. The androgen receptor works synergistically with serum response factor, a negative regulator of Smad3-dependent signaling, to increase muscle differentiation [59], [60]. Androgen receptor is reported to directly bind to and suppress Smad3′s essential role in TGF-β1 signaling [61]. Estrogen also interferes with TGF-β1 signaling; estrogen receptor can bind directly to Smad3 to inhibit its signaling function [62]. Estrogen stimulates muscle repair and regeneration, including the activation and proliferation of satellite cells [63], up-regulates MyoD expression, increases muscle mass and alleviates AP-1-mediated repression of MyoD gene expression [64], [65]. Neither R1881 nor concentrations of estrogen below 10 nM were were sufficient to overcome TGF-β1 inhibition of C2C12 cell muscle differentiation; 10 nM estrogen permitted partial (9%) rescue (p<0.05) (Table 1F).

### Retinoic acid restores C2C12 differentiation in the presence of TGF-β1

Retinoic acid receptors (RARs) heterodimerize with retinoid × receptors (RXRs) in the presence of ligands to regulate a broad array of physiological processes. The heterodimer functions as a transcription factor to regulate gene expression controlling differentiation, cell survival and cell death [66]. Retinoic acid (RA) signaling has important roles in many aspects of development including heart, forelimb, forebrain, hindbrain, pancreas, lung and eye [67], [68]. RA's effects on muscle differentiation are context dependent and include activation of MyoD and myogenin in a rhabdomyosarcoma cell line, repression of Myf5 in myoblasts and inhibition of myogenic differentiation in embryonic limb buds and neonatal limbs [69], [70], [71]. Recently, it was reported that administration of either all-trans RA (atRA) or 9-cisRA induced C2C12 muscle differentiation as assayed using a myosin heavy chain promoter driving Gaussia luciferase, endogenous troponin T expression, endogenous MHC expression and the formation of multi-nucleated fused myoblasts [72]. As these reported studies were done in the absence of exogenous TGF-β1, we examined atRA and 9-cisRA in the presence of 50 pM TGF-β1. Both compounds permitted differentiation of the C2C12 cells in the presence of TGF-β1 comparable to the differentiation obtained with SB431542 and TGF-β1 (Table 1G).

**Figure 4:**
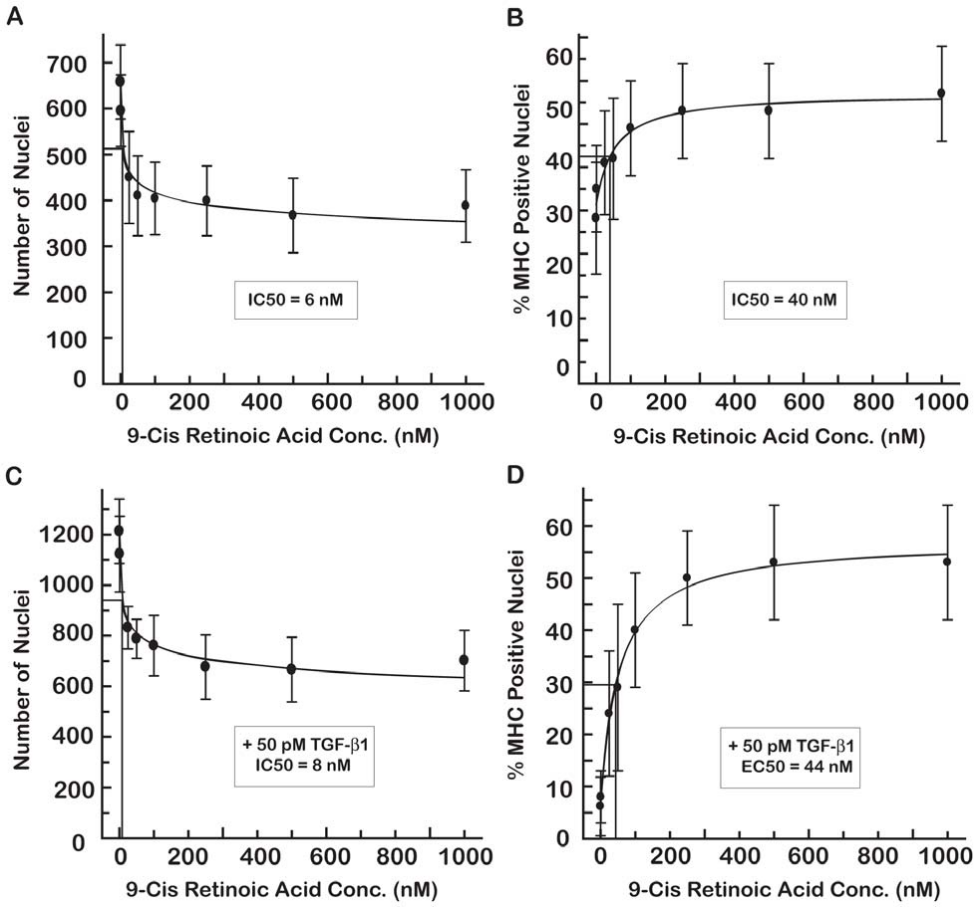
Dose-Response of 9-cisRA on C2C12 proliferation and differentiation in the absence and presence of TGF-β1. 9-cisRA treatment inhibited cell nuclei number with an IC50 of 6 nM (A) and promoted differentiation with an IC50 of 40 nM (B) in the absence of TGF-β1. C2C12 differentiation was inhibited by 50 pM TGF-β1 in panels C and D. 9-cisRA caused a decrease in the number of nuclei with an IC50 of 8 nM (C). 9-cisRA restored myogenesis in the presence of TGF-β1 with an IC50 of 44 nM (D).

**Figure 5:**
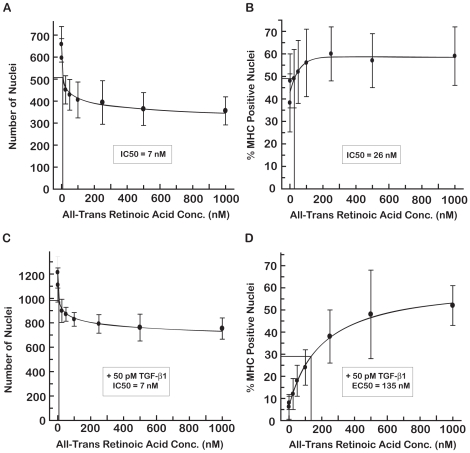
Dose-Response of atRA on C2C12 proliferation and differentiation in the absence and presence of TGF-β1. AtRA treatment inhibited cell nuclei number with an IC50 of 7 nM (A) and promoted differentiation with an IC50 of 26 nM (B) in the absence of TGF-β1. C2C12 differentiation was inhibited by 50 pM TGF-β1 in panels C and D. AtRA caused a decrease in the number of nuclei with an IC50 of 7 nM (C). AtRA restored myogenesis in the presence of TGF-β1 with an IC50 of 135 nM (D).

We used the C2C12 imaging assay to establish the dose-responses of 9-cisRA (Figure 4) and atRA (Figure 5). In the absence of TGF-β1, both RAs caused a reduction of about 30% in the number of nuclei (Figures 4A and 5A). This reduction was less than that caused by SB431542 (Figure 3A) and was quite distinct from the 75–99% reductions in the number of nuclei observed at toxic levels of 17-DMAG (≥100 nM), EGCG (≥125 µM) or resveratrol (≥50 µM). As in the case of SB431542, the reduction in nuclei by 9-cisRA or atRA coincided with increased differentiation of the C2C12 cells (Figures 4B and 5B). The EC50 values for differentiation in the absence of TGF-β1 were 40 nM for 9-cisRA (Figure 4B) and 26 nM for atRA (Figure 5B). The ∼60% increases in percent MHC positive nuclei observed with 9-cisRA and atRA were comparable to the increased differentiation detected in this assay in the presence of 500 µM captopril or 5 nM estrogen in the absence of TGF-β1.

The dose-response of 9-cisRA and atRA on nuclei number and percent MHC positive nuclei was also determined in the presence of TGF-β1. The retinoic acids induced a dose-dependent decrease in the number of nuclei (Figures 4C and 5C) that coincided with an increase in differentiation (Figures 4D and 5D). The EC50 values were 44 nM for 9-cisRA (Figure 4D) and 135 nM for atRA (Figure 5D) for restoring differentiation in the presence of TGF-β1. Although 9-cisRA and atRA induced ∼60% increase in differentiation in the absence of TGF-β1, either RA caused a ∼10× increase in differentiation in the presence of TGF-β1, effectively overcoming the TGF-β1-induced inhibition of myogenesis.

**Figure 6:**
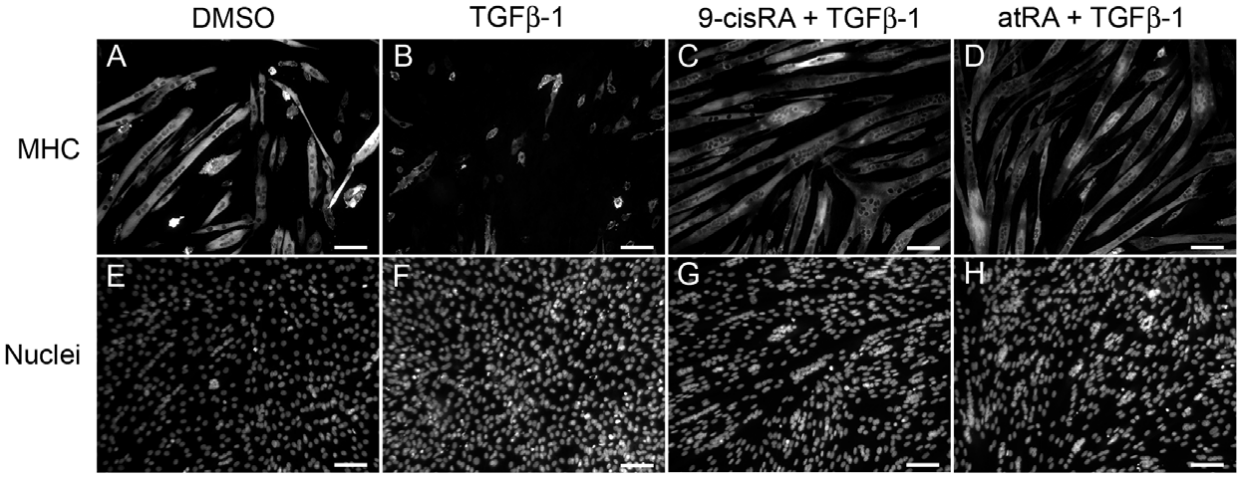
C2C12 muscle differentiation is inhibited by TGF-β1 and restored by 9-cisRA or atRA. Vehicle control had no effect on C2C12 differentiation or cell proliferation (A and E) (scale bar = 100 µm). TGF-β1 inhibited C2C12 myotube formation and increased cell number (B and F). 9-cisRA (500 nM) or atRA (250 nM) restored differentiation of C2C12 cells (C and D) and reduced cell number (G and H) in the presence of TGF-β1.

The effects of the retinoic acids on differentiation could also be observed in the images (Figure 6). Vehicle control did not affect C2C12 differentiation or proliferation (Figure 6A and 6E). TGF-β1 inhibited C2C12 myotube formation and increased the number of nuclei (Figure 6B and 6F). 9-cisRA (500 nM) or atRA (250 nM) permitted differentiation of C2C12 cells in the presence of TGF-β1 (Figure 6C and 6D) and reduced the TGF-β1-induced increase in nuclei (Figure 6G and 6H).

### Vitamin D prevents the retinoic acid restoration of myogenesis in the presence of TGF-β1

**Figure 7:**
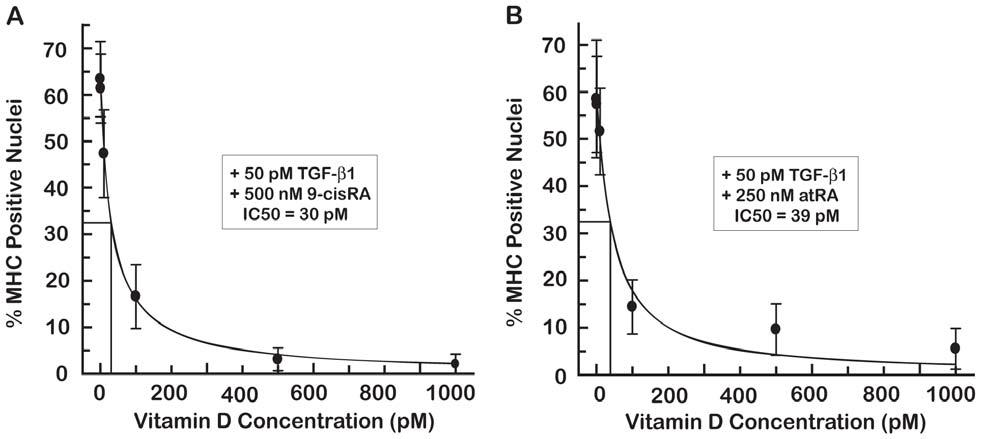
Vitamin D prevents retinoic acid mediated rescue of differentiation in TGF-β1 treated C2C12 cells. C2C12 cells were treated with 50 pM TGF-β1 and either 500 nM 9-cisRA (A) or 250 nM atRA (B) and increasing concentrations of vitamin D for 96 hours. Increasing concentrations of vitamin D reduced the percent of MHC positive nuclei, i.e. myoblast differentiation, with an IC50 of 30 pM in the presence of 9-cisRA (A) or 39 pM in the presence of atRA (B).

There are several examples in which RA and 1α,25-dihydroxyvitamin D_3_ (vitamin D) interact to elucidate a biological response. RA and vitamin D were shown to work synergistically to inhibit neuroblastoma and prostate cancer cell proliferation [73], [74], [75]. In contrast, antagonism between vitamin D and vitamin A, mediated by heterodimerization of ligand bound vitamin D receptors with RXR, also has been reported [76]. In the assay described here, vitamin D alone had a small negative effect on C2C12 cell differentiation in the absence of TGF-β1 and showed evidence of toxicity at 500 pM and 1 nM (data not shown). However, vitamin D was a potent antagonist to 500 nM 9-cisRA (IC50 of 30 pM) and 250 nM atRA (IC50 of 39 pM), as shown by the dose-response to vitamin D in the presence of RA and TGF-β1 (Figure 7A and 7B).

### Retinoic acid does not interfere with TGF-β1-induced Smad3-dependent signaling

Biological interactions between retinoids and TGF-β signaling vary widely and are likely to be highly cell-type and context dependent [77]. RA can have opposing actions on TGF-β1 signaling, either increasing or suppressing TGF-β production and/or signaling depending on cell type, growth conditions and the signaling response examined [77], [78]. For example, atRA suppresses the phosphorylation of Smad2/3 and TGF-β-mediated chondrogenesis [79], [80] and inhibits TGF-β signaling in human lung fibroblast cells [81], [82] and in mesenchymal cells [79]. RA inhibits collagen production in human lung fibroblast cells stimulated with TGF-β1 [82]. In contrast, retinoic acid or analogs increase expression of TGF-β1 and its receptors to facilitate TGF-β-mediated growth inhibition in keratinocytes [83], [84], promyelocytic leukemia cells [85], prostate epithelial cells [86], liver stellate cells [87] and bovine endothelial cells [88]. TGF-β and RA pathways also cooperate to inhibit cell growth in lung epithelial cells [89].

Various molecular mechanisms have been described for the cross-regulation of retinoic acid and TGF-β signaling. atRA reduces the levels of phosphorylated Smad2 and Smad3 in HL60 cells [90]. Direct protein-protein binding interactions between RAR-gamma and Smad3 were reported [81]. In mouse chondrocyte cell cultures, the protein thymine guanine-interacting factor (TGIF) binds to the retinoic response element and Smad2 and Smad3 to interfere with transcriptional activation of the respective pathways; it was proposed that activation of either pathway releases TGIF to inhibit the other pathway [80].

**Figure 8:**
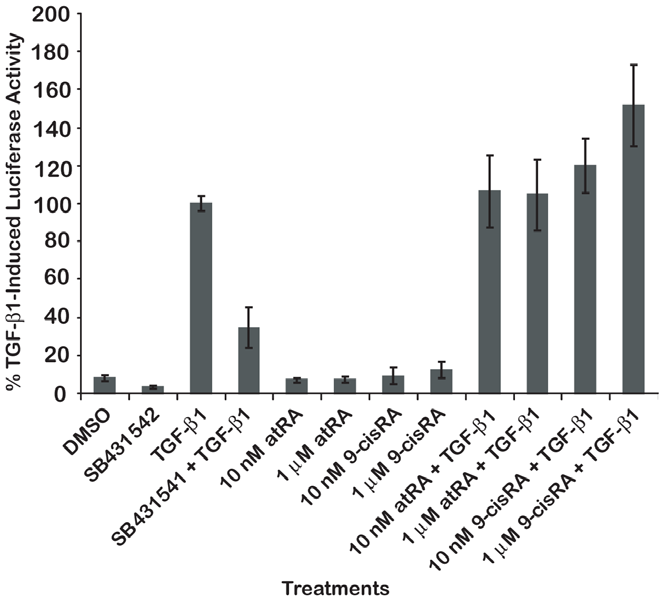
Neither 9-cisRA nor atRA inhibit TGF-β1 induced activation of the Smad3-dependent reporter gene, SBE12-lux. SBE12-lux activity was normalized to treatment with 50 pM TGF-β1 set to 100%. TGF-β1-induced luciferase activity was inhibited by 1 µM SB431542. atRA and 9-cisRA did not inhibit TGF-β1 activation of SBE-lux.

TGF-β1 inhibition of muscle differentiation is mediated through Smad3 which binds directly to the myogenic transcription factors MyoD and MEF2 [9], [10]. To begin to examine the mechanism for RA preventing the TGF-β1 inhibition of muscle differentiation under the conditions of the assay described here, we transfected the TGF-β1-inducible, Smad3-dependent reporter gene SBE12-lux into C2C12 cells (Figure 8). TGF-β1 increased the expression of luciferase from the reporter. This increase was fully inhibited by 1 µM SB431542, but was not altered by the presence of 10 nM or 1 µM atRA or 9-cisRA. As activation of SBE12-lux requires proper activity of the receptors for TGF-β1 and the Smad3 and Smad4 proteins, this result suggests that atRA is acting downstream of these components of TGF-β1 signaling to overcome the anti-myogenic effect of TGF-β1.

### Conclusions

We report a cell-based assay using high content imaging to quantify myoblast differentiation by the presence of MHC expression surrounding each nucleus in the field. The specific conditions chosen here precluded detecting some agents that accelerate C2C12 differentiation into multinucleate myotubes in the absence of TGF-β1; however, the assay conditions were optimized to detect agents that overcome the effect of TGF-β1 on C2C12 differentiation. As with any *in vitro* screening assay, C2C12 differentiation will miss some agents that are active *in vivo*; the inability of losartan to be detected in the assay provides an example of this limitation.

Of the 13 agents tested, only atRA and 9-cisRA were effective in fully restoring C2C12 differentiation in the presence of TGF-β1. The identification of RAs is intriguing in that some retinoids are already approved for human therapy, most notably for the treatment of acute promyelocytic leukemia [91] and some skin disorders [92]. Further studies are needed to determine if RAs ameliorate symptoms in muscle disease that are due to poor muscle regeneration caused by high constitutive levels of TGF-β1. Also, it is important to note that there also are well-known adverse effects of using retinoids in humans. The two most commonly prescribed oral retinoids, isotretinoin and acitretin, are associated with generalized muscle stiffness syndrome and myalgia in some individuals [93]. Other adverse effects of oral retinoids include dryness of mucous membranes and eyes, increase in serum triglycerides and teratogenicity [94]. So far, the RXR ligands, rexinoids, appear to be less toxic, i.e., the major side-effect of bexarotene/Targretin is hyperglyceridaemia [95], [96], [97]. A recent review lists 11 retinoids, 2 rexinoids and 4 atypical retinoids in clinical trials or approved for therapy [66]. The quantitative assay described here will be useful for evaluating less-toxic retinoids and also combinations of agents that may permit lower doses of retinoids to be effective in allowing myoblast differentiation in the presence of TGF-β1.

## References

[1] Shi Y, Massague J (2003). Mechanisms of TGF-beta signaling from cell membrane to the nucleus.. Cell.

[2] ten Dijke P, Hill CS (2004). New insights into TGF-beta-Smad signalling.. Trends Biochem Sci.

[3] Sporn MB, Roberts AB (1990). TGF-beta: problems and prospects.. Cell Regul.

[4] Siegel PM, Massague J (2003). Cytostatic and apoptotic actions of TGF-beta in homeostasis and cancer.. Nat Rev Cancer.

[5] Rosenbloom J, Castro SV, Jimenez SA (2010). Narrative review: fibrotic diseases: cellular and molecular mechanisms and novel therapies.. Ann Intern Med.

[6] Massague J, Cheifetz S, Endo T, Nadal-Ginard B (1986). Type beta transforming growth factor is an inhibitor of myogenic differentiation.. Proc Natl Acad Sci U S A.

[7] Olson EN, Sternberg E, Hu JS, Spizz G, Wilcox C (1986). Regulation of myogenic differentiation by type beta transforming growth factor.. J Cell Biol.

[8] Brennan TJ, Edmondson DG, Li L, Olson EN (1991). Transforming growth factor beta represses the actions of myogenin through a mechanism independent of DNA binding.. Proc Natl Acad Sci U S A.

[9] Liu D, Black BL, Derynck R (2001). TGF-beta inhibits muscle differentiation through functional repression of myogenic transcription factors by Smad3.. Genes Dev.

[10] Liu D, Kang JS, Derynck R (2004). TGF-beta-activated Smad3 represses MEF2-dependent transcription in myogenic differentiation.. EMBO J.

[11] Kollias HD, Perry RL, Miyake T, Aziz A, McDermott JC (2006). Smad7 promotes and enhances skeletal muscle differentiation.. Mol Cell Biol.

[12] McPherron AC, Lawler AM, Lee SJ (1997). Regulation of skeletal muscle mass in mice by a new TGF-beta superfamily member.. Nature.

[13] Amthor H, Huang R, McKinnell I, Christ B, Kambadur R (2002). The regulation and action of myostatin as a negative regulator of muscle development during avian embryogenesis.. Dev Biol.

[14] Bernasconi P, Di Blasi C, Mora M, Morandi L, Galbiati S (1999). Transforming growth factor-beta1 and fibrosis in congenital muscular dystrophies.. Neuromuscul Disord.

[15] Gosselin LE, Williams JE, Deering M, Brazeau D, Koury S (2004). Localization and early time course of TGF-beta 1 mRNA expression in dystrophic muscle.. Muscle Nerve.

[16] Desmouliere A, Geinoz A, Gabbiani F, Gabbiani G (1993). Transforming growth factor-beta 1 induces alpha-smooth muscle actin expression in granulation tissue myofibroblasts and in quiescent and growing cultured fibroblasts.. J Cell Biol.

[17] Tomasek JJ, Gabbiani G, Hinz B, Chaponnier C, Brown RA (2002). Myofibroblasts and mechano-regulation of connective tissue remodelling.. Nat Rev Mol Cell Biol.

[18] Li Y, Foster W, Deasy BM, Chan Y, Prisk V (2004). Transforming growth factor-beta1 induces the differentiation of myogenic cells into fibrotic cells in injured skeletal muscle: a key event in muscle fibrogenesis.. Am J Pathol.

[19] Li Y, Huard J (2002). Differentiation of muscle-derived cells into myofibroblasts in injured skeletal muscle.. Am J Pathol.

[20] Cohn RD, van Erp C, Habashi JP, Soleimani AA, Klein EC (2007). Angiotensin II type 1 receptor blockade attenuates TGF-beta-induced failure of muscle regeneration in multiple myopathic states.. Nat Med.

[21] Dezawa M, Ishikawa H, Itokazu Y, Yoshihara T, Hoshino M (2005). Bone marrow stromal cells generate muscle cells and repair muscle degeneration.. Science.

[22] Peault B, Rudnicki M, Torrente Y, Cossu G, Tremblay JP (2007). Stem and progenitor cells in skeletal muscle development, maintenance, and therapy.. Mol Ther.

[23] Reimann J, Irintchev A, Wernig A (2000). Regenerative capacity and the number of satellite cells in soleus muscles of normal and mdx mice.. Neuromuscul Disord.

[24] Lavoie P, Robitaille G, Agharazii M, Ledbetter S, Lebel M (2005). Neutralization of transforming growth factor-beta attenuates hypertension and prevents renal injury in uremic rats.. J Hypertens.

[25] Lim DS, Lutucuta S, Bachireddy P, Youker K, Evans A (2001). Angiotensin II blockade reverses myocardial fibrosis in a transgenic mouse model of human hypertrophic cardiomyopathy.. Circulation.

[26] Sugaru E, Nakagawa T, Ono-Kishino M, Nagamine J, Tokunaga T (2006). Enhanced effect of combined treatment with SMP-534 (antifibrotic agent) and losartan in diabetic nephropathy.. Am J Nephrol.

[27] Sugaru E, Nakagawa T, Ono-Kishino M, Nagamine J, Tokunaga T (2007). Amelioration of established diabetic nephropathy by combined treatment with SMP-534 (antifibrotic agent) and losartan in db/db mice.. Nephron Exp Nephrol.

[28] Yu L, Border WA, Anderson I, McCourt M, Huang Y (2004). Combining TGF-beta inhibition and angiotensin II blockade results in enhanced antifibrotic effect.. Kidney Int.

[29] Yingling JM, Blanchard KL, Sawyer JS (2004). Development of TGF-beta signalling inhibitors for cancer therapy.. Nat Rev Drug Discov.

[30] Inman GJ, Nicolas FJ, Callahan JF, Harling JD, Gaster LM (2002). SB-431542 is a potent and specific inhibitor of transforming growth factor-beta superfamily type I activin receptor-like kinase (ALK) receptors ALK4, ALK5, and ALK7.. Mol Pharmacol.

[31] Watt KI, Jaspers RT, Atherton P, Smith K, Rennie MJ (2010). SB431542 treatment promotes the hypertrophy of skeletal muscle fibers but decreases specific force..

[32] Garber K (2009). Companies waver in efforts to target transforming growth factor beta in cancer.. J Natl Cancer Inst.

[33] Dietz HC (2010). TGF-beta in the pathogenesis and prevention of disease: a matter of aneurysmic proportions.. J Clin Invest.

[34] Portier GL, Benders AG, Oosterhof A, Veerkamp JH, van Kuppevelt TH (1999). Differentiation markers of mouse C2C12 and rat L6 myogenic cell lines and the effect of the differentiation medium.. In Vitro Cell Dev Biol Anim.

[35] Mori S, Tokuyama K (2007). Variation in ACE activity affects myogenic differentiation in C2C12 cells.. Biochem Biophys Res Commun.

[36] De Albuquerque DA, Saxena V, Adams DE, Boivin GP, Brunner HI (2004). An ACE inhibitor reduces Th2 cytokines and TGF-beta1 and TGF-beta2 isoforms in murine lupus nephritis.. Kidney Int.

[37] Wengrower D, Zanninelli G, Pappo O, Latella G, Sestieri M (2004). Prevention of fibrosis in experimental colitis by captopril: the role of tgf-beta1.. Inflamm Bowel Dis.

[38] Mori S, Tokuyama K (2007). ACE activity affects myogenic differentiation via mTOR signaling.. Biochem Biophys Res Commun.

[39] Izumi K, Mizokami A, Li YQ, Narimoto K, Sugimoto K (2009). Tranilast inhibits hormone refractory prostate cancer cell proliferation and suppresses transforming growth factor beta1-associated osteoblastic changes.. Prostate.

[40] Chakrabarti R, Subramaniam V, Abdalla S, Jothy S, Prud'homme GJ (2009). Tranilast inhibits the growth and metastasis of mammary carcinoma.. Anticancer Drugs.

[41] Iwata Y, Katanosaka Y, Shijun Z, Kobayashi Y, Hanada H (2005). Protective effects of Ca2+ handling drugs against abnormal Ca2+ homeostasis and cell damage in myopathic skeletal muscle cells.. Biochem Pharmacol.

[42] Jinnin M, Ihn H, Tamaki K (2006). Characterization of SIS3, a novel specific inhibitor of Smad3, and its effect on transforming growth factor-beta1-induced extracellular matrix expression.. Mol Pharmacol.

[43] Li ZB, Kollias HD, Wagner KR (2008). Myostatin directly regulates skeletal muscle fibrosis.. J Biol Chem.

[44] Yun BG, Matts RL (2005). Hsp90 functions to balance the phosphorylation state of Akt during C2C12 myoblast differentiation.. Cell Signal.

[45] Yun CH, Yoon SY, Nguyen TT, Cho HY, Kim TH (2010). Geldanamycin inhibits TGF-beta signaling through induction of Hsp70.. Arch Biochem Biophys.

[46] Wrighton KH, Lin X, Feng XH (2008). Critical regulation of TGFbeta signaling by Hsp90.. Proc Natl Acad Sci U S A.

[47] Heeg MH, Koziolek MJ, Vasko R, Schaefer L, Sharma K (2005). The antifibrotic effects of relaxin in human renal fibroblasts are mediated in part by inhibition of the Smad2 pathway.. Kidney Int.

[48] Negishi S, Li Y, Usas A, Fu FH, Huard J (2005). The effect of relaxin treatment on skeletal muscle injuries.. Am J Sports Med.

[49] Samuel CS (2005). Relaxin: antifibrotic properties and effects in models of disease.. Clin Med Res.

[50] Messina S, Bitto A, Aguennouz M, Mazzeo A, Migliorato A (2009). Flavocoxid counteracts muscle necrosis and improves functional properties in mdx mice: a comparison study with methylprednisolone.. Exp Neurol.

[51] Brisdelli F, D'Andrea G, Bozzi A (2009). Resveratrol: a natural polyphenol with multiple chemopreventive properties.. Curr Drug Metab.

[52] Hemdan DI, Hirasaka K, Nakao R, Kohno S, Kagawa S (2009). Polyphenols prevent clinorotation-induced expression of atrogenes in mouse C2C12 skeletal myotubes.. J Med Invest.

[53] Park CE, Kim MJ, Lee JH, Min BI, Bae H (2007). Resveratrol stimulates glucose transport in C2C12 myotubes by activating AMP-activated protein kinase.. Exp Mol Med.

[54] Dirks Naylor AJ (2009). Cellular effects of resveratrol in skeletal muscle.. Life Sci.

[55] Wyke SM, Russell ST, Tisdale MJ (2004). Induction of proteasome expression in skeletal muscle is attenuated by inhibitors of NF-kappaB activation.. Br J Cancer.

[56] Nakae Y, Hirasaka K, Goto J, Nikawa T, Shono M (2008). Subcutaneous injection, from birth, of epigallocatechin-3-gallate, a component of green tea, limits the onset of muscular dystrophy in mdx mice: a quantitative histological, immunohistochemical and electrophysiological study.. Histochem Cell Biol.

[57] Brown D, Hikim AP, Kovacheva EL, Sinha-Hikim I (2009). Mouse model of testosterone-induced muscle fiber hypertrophy: involvement of p38 mitogen-activated protein kinase-mediated Notch signaling.. J Endocrinol.

[58] Kovacheva EL, Hikim AP, Shen R, Sinha I, Sinha-Hikim I Testosterone supplementation reverses sarcopenia in aging through regulation of myostatin, c-Jun NH2-terminal kinase, Notch, and Akt signaling pathways.. Endocrinology.

[59] Vlahopoulos S, Zimmer WE, Jenster G, Belaguli NS, Balk SP (2005). Recruitment of the androgen receptor via serum response factor facilitates expression of a myogenic gene.. J Biol Chem.

[60] Lee HJ, Yun CH, Lim SH, Kim BC, Baik KG (2007). SRF is a nuclear repressor of Smad3-mediated TGF-beta signaling.. Oncogene.

[61] Chipuk JE, Cornelius SC, Pultz NJ, Jorgensen JS, Bonham MJ (2002). The androgen receptor represses transforming growth factor-beta signaling through interaction with Smad3.. J Biol Chem.

[62] Matsuda T, Yamamoto T, Muraguchi A, Saatcioglu F (2001). Cross-talk between transforming growth factor-beta and estrogen receptor signaling through Smad3.. J Biol Chem.

[63] Enns DL, Tiidus PM (2010). The influence of estrogen on skeletal muscle: sex matters.. Sports Med.

[64] Pedraza-Alva G, Zingg JM, Donda A, Perez-Martinez L (2009). Estrogen receptor regulates MyoD gene expression by preventing AP-1-mediated repression.. Biochem Biophys Res Commun.

[65] Kahlert S, Grohe C, Karas RH, Lobbert K, Neyses L (1997). Effects of estrogen on skeletal myoblast growth.. Biochem Biophys Res Commun.

[66] Altucci L, Leibowitz MD, Ogilvie KM, de Lera AR, Gronemeyer H (2007). RAR and RXR modulation in cancer and metabolic disease.. Nat Rev Drug Discov.

[67] Duester G (2008). Retinoic acid synthesis and signaling during early organogenesis.. Cell.

[68] Niederreither K, Dolle P (2008). Retinoic acid in development: towards an integrated view.. Nat Rev Genet.

[69] Arnold HH, Gerharz CD, Gabbert HE, Salminen A (1992). Retinoic acid induces myogenin synthesis and myogenic differentiation in the rat rhabdomyosarcoma cell line BA-Han-1C.. J Cell Biol.

[70] Carnac G, Albagli-Curiel O, Levin A, Bonnieu A (1993). 9-cis-retinoic acid regulates the expression of the muscle determination gene Myf5.. Endocrinology.

[71] Xiao Y, Grieshammer U, Rosenthal N (1995). Regulation of a muscle-specific transgene by retinoic acid.. J Cell Biol.

[72] Zhu GH, Huang J, Bi Y, Su Y, Tang Y (2009). Activation of RXR and RAR signaling promotes myogenic differentiation of myoblastic C2C12 cells.. Differentiation.

[73] Stio M, Celli A, Treves C (2001). Synergistic anti-proliferative effects of vitamin D derivatives and 9-cis retinoic acid in SH-SY5Y human neuroblastoma cells.. J Steroid Biochem Mol Biol.

[74] Stio M, Celli A, Treves C (2002). Synergistic effect of vitamin D derivatives and retinoids on C2C12 skeletal muscle cells.. IUBMB Life.

[75] Blutt SE, Allegretto EA, Pike JW, Weigel NL (1997). 1,25-dihydroxyvitamin D3 and 9-cis-retinoic acid act synergistically to inhibit the growth of LNCaP prostate cells and cause accumulation of cells in G1.. Endocrinology.

[76] Bastie JN, Balitrand N, Guidez F, Guillemot I, Larghero J (2004). 1 alpha,25-dihydroxyvitamin D3 transrepresses retinoic acid transcriptional activity via vitamin D receptor in myeloid cells.. Mol Endocrinol.

[77] Roberts AB, Sporn MB (1992). Mechanistic interrelationships between two superfamilies: the steroid/retinoid receptors and transforming growth factor-beta.. Cancer Surv.

[78] Mucida D, Cheroutre H (2007). TGFbeta and retinoic acid intersect in immune-regulation.. Cell Adh Migr.

[79] Yu Z, Xing Y (2006). All-trans retinoic acid inhibited chondrogenesis of mouse embryonic palate mesenchymal cells by down-regulation of TGF-beta/Smad signaling.. Biochem Biophys Res Commun.

[80] Zhang H, Li N, Tang Y, Wu W, Zhang Q (2009). Negative functional interaction of retinoic acid and TGF-beta signaling mediated by TG-interacting factor during chondrogenesis.. Cell Physiol Biochem.

[81] Pendaries V, Verrecchia F, Michel S, Mauviel A (2003). Retinoic acid receptors interfere with the TGF-beta/Smad signaling pathway in a ligand-specific manner.. Oncogene.

[82] Redlich CA, Delisser HM, Elias JA (1995). Retinoic acid inhibition of transforming growth factor-beta-induced collagen production by human lung fibroblasts.. Am J Respir Cell Mol Biol.

[83] Glick AB, Flanders KC, Danielpour D, Yuspa SH, Sporn MB (1989). Retinoic acid induces transforming growth factor-beta 2 in cultured keratinocytes and mouse epidermis.. Cell Regul.

[84] Borger DR, Mi Y, Geslani G, Zyzak LL, Batova A (2000). Retinoic acid resistance at late stages of human papillomavirus type 16-mediated transformation of human keratinocytes arises despite intact retinoid signaling and is due to a loss of sensitivity to transforming growth factor-beta.. Virology.

[85] Nunes I, Kojima S, Rifkin DB (1996). Effects of endogenously activated transforming growth factor-beta on growth and differentiation of retinoic acid-treated HL-60 cells.. Cancer Res.

[86] Danielpour D (1996). Induction of transforming growth factor-beta autocrine activity by all-trans-retinoic acid and 1 alpha,25-dihydroxyvitamin D3 in NRP-152 rat prostatic epithelial cells.. J Cell Physiol.

[87] Imai S, Okuno M, Moriwaki H, Muto Y, Murakami K (1997). 9,13-di-cis-Retinoic acid induces the production of tPA and activation of latent TGF-beta via RAR alpha in a human liver stellate cell line, LI90.. FEBS Lett.

[88] Kojima S, Rifkin DB (1993). Mechanism of retinoid-induced activation of latent transforming growth factor-beta in bovine endothelial cells.. J Cell Physiol.

[89] La P, Morgan TA, Sykes SM, Mao H, Schnepp RW (2003). Fusion proteins of retinoid receptors antagonize TGF-beta-induced growth inhibition of lung epithelial cells.. Oncogene.

[90] Cao Z, Flanders KC, Bertolette D, Lyakh LA, Wurthner JU (2003). Levels of phospho-Smad2/3 are sensors of the interplay between effects of TGF-beta and retinoic acid on monocytic and granulocytic differentiation of HL-60 cells.. Blood.

[91] Lengfelder E, Saussele S, Weisser A, Buchner T, Hehlmann R (2005). Treatment concepts of acute promyelocytic leukemia.. Crit Rev Oncol Hematol.

[92] Thielitz A, Krautheim A, Gollnick H (2006). Update in retinoid therapy of acne.. Dermatol Ther.

[93] Chroni E, Monastirli A, Tsambaos D (2010). Neuromuscular adverse effects associated with systemic retinoid dermatotherapy: monitoring and treatment algorithm for clinicians.. Drug Saf.

[94] Lens M, Medenica L (2008). Systemic retinoids in chemoprevention of non-melanoma skin cancer.. Expert Opin Pharmacother.

[95] Miller VA, Benedetti FM, Rigas JR, Verret AL, Pfister DG (1997). Initial clinical trial of a selective retinoid X receptor ligand, LGD1069.. J Clin Oncol.

[96] Kempf W, Kettelhack N, Duvic M, Burg G (2003). Topical and systemic retinoid therapy for cutaneous T-cell lymphoma.. Hematol Oncol Clin North Am.

[97] Zhang C, Duvic M (2003). Retinoids: therapeutic applications and mechanisms of action in cutaneous T-cell lymphoma.. Dermatol Ther.

